# Experience of office-based haematologists and oncologists with outpatient psycho-social support services for cancer patients with and without migration background in Germany

**DOI:** 10.1007/s00432-022-04313-6

**Published:** 2022-09-02

**Authors:** Nicola Riccetti, Isabelle Hempler, Kerstin Hermes-Moll, Vitali Heidt, Thomas Walawgo, Susanne Singer

**Affiliations:** 1 Institute of Medical Biostatistics, Epidemiology and Informatics (IMBEI), University Medical Centre, Obere Zahlbacher Str. 69, 55131 Mainz, Germany; 2 University Cancer Centre Mainz, Mainz, Germany; 3 Scientific Institute of Office-Based Haematologists and Oncologists (WINHO GmbH), Cologne, Germany

**Keywords:** Psycho-oncology, Referral and consultation, Medical oncology, Physician–patient relations, Neoplasm

## Abstract

**Purpose:**

We compared the perception of office-based haematologists and oncologists regarding the availability of outpatient psycho-social support services (PSSS) for patients with cancer and a migration background, as well as their different experiences with these services.

**Methods:**

Data were collected via an online survey addressing the doctors’ socio-demographic characteristics and their perception of-and experience with PSSS. The association between socio-demographic characteristics of the doctors and their experiences with PSSS was tested using Pearson’s chi-squared test and Kruskal–Wallis test.

**Results:**

A total of 55 doctors were included in this study. More than three doctors in four reported non-sufficient presence of PSSS in foreign languages in their region; one in two reported that the services for patients with migration background should be improved. Most doctors reported missing PSSS in Turkish and Arabic in their region.

Doctors with less experience referred patients more often to PSSS hosted in patients’ associations (75% vs 25%; *p* = 0.02), than doctors with more experience. Doctors working in larger cities referred patients less often to PSSS in cancer counselling centres (12% vs 88%; *p* = 0.01), than doctors working in small or middle-large cities. Male doctors were more satisfied with the network of PSSS’ providers, than female doctors (mean score = 2.8 vs 2.2; *p* = 0.05).

**Conclusions:**

Our results suggest that efforts should be made for a higher regional availability of overall and specific PSSS for non-German speaking patients (especially for Turkish- and Arabic-speaking patients). The experience with PSSS was associated with the doctors’ work experience and gender, as well as the location of the practice.

## Introduction

Patients with cancer who uptake psycho-social support services improve their quality of life, emotional and social function, and reduce distress, depression and anxiety (Buffart et al. 2020; Goodwin et al. 2001; Kalter et al. 2018; Marchioro et al. 1996; Warth et al. 2020). For these reasons, psycho-social support services are an integral part of cancer care in many countries, and are available in both in- and outpatient facilities (Herschbach and Mandel 2011).

However, despite their efficacy and growing availability, the use of psycho-social support services especially in the outpatient setting is lower than the patients’ need for it (Faller et al. 2017; Frey Nascimento et al. 2019; Singer et al. 2013a, b). Inequalities in the availability of the services might create barriers to the participation. For instance, the services are more available in large certified structures (Certified Cancer Centres [CCC]), which are rare in rural areas (Kowalski et al. 2016; Singer et al. 2013a): Singer et al. (2012) reported that half of the facilities providing outpatient psycho-social support services were in cities between 20,000 and 100,000 inhabitants, and only around one-third and one-sixth were in smaller or larger cities, respectively. In addition, these structures were often combined with barriers to the accessibility, e.g. lack of parking facilities or of transport connections.

Another barrier to the participation in psycho-social support services is the lack of the doctors’ recommendation (Eakin and Strycker 2001; Frey Nascimento et al. 2019). Patients with cancer who receive a recommendation from their oncologist are six-times more likely to take up psycho-social support services compared to patients with no recommendation. Conversely, no association is present between the uptake of the services and the level of details of the information provided (Frey Nascimento et al. 2019).

Therefore, to enhance the participation in psycho-social support services, an increase in referrals by doctors might be beneficial. To achieve this, it is important that  the respective services are available in close proximity to the practices and patients, and that doctors and their patients have positive experiences with the service providers.

No conclusive information is available on whether participation in psycho-social support services varies based on the migration background of the patients in Germany (Singer et al. 2022; Zeissig et al. 2015). However, patients with cancer and migration background are considered to be more likely to have higher psycho-social difficulties (Riccetti et al. 2022a; Tibubos et al. 2018), as well as higher barriers to access supportive care services than non-migrant patients (Riccetti et al. 2022a, b; Riccetti et al. 2020; Sze et al. 2015). Linguistically and culturally competent psycho-social support services are not available in every region (Schulz et al. 2018), often leading to friends and/or relatives acting as translators, with further difficulties and barriers for patients in discussing their need for psycho-social support (Hermes-Moll et al. 2022a).

The aim of this study is, therefore, to investigate the availability of- and the experience with psycho-social support services for cancer patients with and without migration background from the perspective of office-based haematologists and oncologists in Germany, comparing different groups of doctors based on their socio-demographic characteristics and on the characteristics of the practices they work in.

The study questions are:

- Is the regional availability of overall and specific psycho-social support services for non-German speaking patients considered to be sufficient by office-based haematologists and oncologists in Germany?
- Does the experience of office-based haematologists and oncologists with psycho-social support services for cancer patients with and without migration background in Germany differ based on their socio-demographic characteristics and on the characteristics of the practices they work in?

## Methods

### Study design

Data collection took place between December 2020 and March 2021 via an anonymous, nation-wide online survey. The survey was part of the mixed-methods study “Psycho-oncological support in cancer patients with migration background” (*Psychoonkologische Versorgung von Krebspatienten mit Migrationshintergund* [POM]), which was described in detail for its purposes and results elsewhere (Hempler et al. 2021a, b, c; Hermes-Moll et al. 2022b).

An email invitation to participate in the survey was sent to 581 doctors in 380 haematology and oncology practices in the networks of the Scientific Institute of Office-based Haematologists and Oncologists (*Wissenschaftliches Institut der Niedergelassenen Hämatologen und Onkologen* [WINHO]) and of the Professional Association of Office-based Haematologists and Oncologists in Germany (*Berufsverband der Niedergelassenen Hämatologen und Onkologen in Deutschland* [BNHO]). On January 2021, a second email was sent to all the practices, as reminder of the participation in the survey.

This study obtained ethical approval from the Rhineland-Palatinate State Medical Association (2019–14,424).

### Survey

The survey was developed following a series of qualitative interviews with office-based haematologists and oncologists conducted in a previous stage of the project, and described in detail elsewhere (Hempler et al. 2021a, b).

It comprised sections for: (a) socio-demographic characteristics of the doctors, (b) communication with patients with migration background, (c) cultural differences in patients with migration background, (d) experiences with screening instruments for psychological distress, (e) role of relatives and caregivers, and (f) experiences with psycho-social support services.

#### Operationalization of the variables

This analysis focuses on the results of the section of the survey on the experience with psycho-social support services. The section comprised:

1. The presence and characteristics of psycho-social support services in the practices. It included the presence, amount and type of psycho-social services in the practice (presence: yes/no/missing; hours: 40 h/week, 20–39 h/week, 10–19 h/week, 5–9 h/week, less than 5 h/week/missing; type of worker: social worker/psychologist/other/missing);
2. The opinion of the doctors on whether psycho-social support services in the practice was an advantage, and whether psycho-social support services for migrant patients should be improved (both: yes/no/missing);
3. The psycho-social support service(s) to which the doctors had referred their patients to (office-based psychotherapist/psycho-social cancer counselling centre/self-help group/patients association/outpatient clinics/no possibility/other/missing);
4. The satisfaction with the availability of psycho-social support services in the region, both overall and specifically for patients with migration background, considered as services in languages other than German (both: yes/no/missing);
5. Missing languages among the psycho-social support services provided in the region; and
6. The satisfaction with the network and organization of and the connection with the outpatient psycho-social support services providers. Variables in this section were recoded as three continuous variables, ranging from “not at all satisfied” (1) to “completely satisfied” (4).

The following socio-demographic characteristics of the doctors were ascertained: gender (male/female/other), age (under 49/50–59/60 or more), years of work experience (5–10/11–20/more than 20), country of birth (*free-text*), further education in psycho-oncology (yes/no), languages spoken other than German (one/ two/ three or more/ no foreign languages or missing), type of practice (single practice [*Einzelpraxis*]/joint practice [*Gemeinschaftspraxis*]/medical care centre [*Medizinisches Versorgungszentrum—MVZ*]), location of the practice (large city [≥ 100,000 inhabitants]/middle-size city [between ≥ 20,000 and < 100,000 inhabitants]/small city [between ≥5,000 and < 20,000 inhabitants]/village [< 5,000 inhabitants]).

### Statistical analysis

We reported the experiences with outpatient psycho-social support services both overall, as well as stratified by socio-demographic characteristics of the doctors and of the practices.

We used Pearson’s chi-squared tests for categorical data and Kruskal–Wallis tests for continuous data to explore the univariate association of socio-demographic characteristics of the doctors and characteristics of the practices they work in with their experience with outpatient psycho-social support services. To avoid over dispersion, in the group comparison the listed variables were recoded as follows: years of age (< 60 years/ ≥ 60 years), years of work experience (< 20 years/ ≥ 20 years), country of birth (Germany/other), type of practice (joint practice vs individual practices and medical care centres), location of the practice (large city vs small city, middle-large city, and village).

## Results

### Sample description

Of the 581 doctors contacted, 55 (9%) completed at least 5% of the questionnaire and were included in this analysis.

Doctors were mostly male (65%), older than 50 years old (73%), with more than 20 years of work experience (67%), born in Germany (85%) and working in cities with 100,000 inhabitants or more (65%). When asked about their proficiency with foreign languages, 40% of the doctors reported speaking one, 9% reported speaking two, and 5% reported speaking three or more (Table 1).

**Table 1:** Socio-demographic characteristics of the doctors in the study sample and general characteristics of the practices they work in (*N* = 55)

Variables	*N*	%
Gender		
Female	13	23.6
Male	36	65.5
Missing	6	10.9
Age		
Under 50 years	10	18.2
50–59 years	24	43.6
60 years or over	16	29.1
Missing	5	9.1
Work experience		
5–11 years	4	7.3
11–20 years	10	18.2
Over 20 years	37	67.3
Missing	4	7.3
Country of birth		
Germany	47	85.4
Other/Missing	8	14.6
Psycho-oncological further education		
No	39	70.9
Yes	11	20.0
Missing	5	9.1
Foreign languages spoken		
One foreign language	22	40.0
Two foreign languages	5	9.1
Three or more foreign languages	3	5.4
No foreign language/Missing	25	45.5
Type of practice		
Individual practice	5	9.1
Joint practice	38	69.1
Medical care centres	8	14.6
Missing	4	7.3
Location of the practice		
Large city (≥ 100,000 inhabitants)	36	65.5
Middle-large city (≥ 20,000 and < 100,000 inhabitants)	12	21.8
Small city (≥ 5,000 and < 20,000 inhabitants)	4	7.3
Missing	3	5.5

### Experience with psycho-social support services

A total of 29 doctors (53%) reported referring patients to outpatient psychotherapists, 33 (60%) to psycho-social cancer counselling centres, 13 (24%) to self-help groups, and 4 (7%) each to patients’ associations, outpatient clinics and other unspecified services.

Among the doctors, 38 (69%) did not have a psycho-social support service directly in their practices, whereas 39 (71%) of them reported considering it an advantage. More than half of the respondents (56%) reported a non-sufficient presence of overall psycho-social support services in their region.

On a scale from 1 (lower satisfaction) to 4 (higher satisfaction), the average reported satisfaction with network, organization and cooperation was 2.6 (standard deviation [SD] = 1.1), 2.3 (SD = 1.1), and 2.5 (SD 1.1), respectively (Table 2).

**Table 2:** The presence and form of psycho-social support service in the practice, the availability of psycho-social support services in the region overall and specifically for patients with migration background, and the satisfaction with network, organization and cooperation with psycho-social support services and services providers (*N* = 55)

Variables	*N*	%
Psycho-social support service in the practice		
No	38	69.1
Yes	14	25.5
Missing	3	5.5
If yes, how many hours		
40 h/week	1	1.8
20–39 h/week	3	5.4
10–19 h/week	6	10.9
5–9 h/week	3	5.4
Less than 5 h/week	6	10.9
Missing	36	65.5
Which work-group		
Social worker	6	10.9
Psychologist	5	9.1
Other	6	10.9
Missing	38	69.1
Psycho-social support service in practice as advantage		
No	13	23.6
Yes	39	70.9
Missing	3	5.5
Sufficient availability of psycho-social support service in the region		
No	31	56.4
Yes	21	38.2
Missing	3	5.5
Sufficient availability of psycho-social support service in foreign language in the region		
No	44	80
Yes	1	1.8
Missing	10	18.2
Psycho-social support service for patients with migration background can be improved		
No	28	50.9
Yes	24	43.6
Missing	3	5.5

	*N*	Mean	SD
Satisfaction with network^a^	51	2.6	1.1
Satisfaction with organization^a^	51	2.3	1.1
Satisfaction with cooperation^a^	49	2.5	1.1

^a^Scale between 1 (not at all satisfied) and 4 (completely satisfied)

### Psycho-social support services for patients with migration background

More than three doctors in four (80%) reported non-sufficient availability of psycho-social support services in foreign language in their region, and almost one in two (44%) reported that psycho-social support services for patients with migration background can be improved (Tables 2, 3).

**Table 3:** Availability of psycho-social support services overall and in foreign language, and opinion on whether psycho-social support services (PSSS) for patients with migration background can be improved by socio-demographic characteristics of the doctors and characteristics of the practices they work in (*N* = 55)

Covariates	Sufficient availability of PSSS			Sufficient availability of PSSS in foreign languages			PSSS for patients with migration background can be improved		
	Yes		*p*	Yes		*p*	Yes		*p*
	*N*	%		*N*	%		*N*	%	
Gender									
Female	5	23.8	0.78	0	0	0.52	6	25	0.84
Male	15	71.4		1	100		15	62.5	
Years of age									
< 60 years	13	61.9	0.66	1	100	0.52	17	70.8	0.44
≥ 60 years	8	38.1		0	0		7	29.2	
Years of work experience									
< 20 years	4	19.1	0.29	1	100	0.11	8	33.3	0.33
≥ 20 years	17	81		0	0		16	66.7	
Country of birth									
Germany	19	90.5	0.71	1	100	0.69	21	87.5	0.84
Other/Missing	2	9.5		0	0		3	12.5	
Psycho-oncological further education									
No	14	66.7	0.10	1	100	0.60	20	83.3	0.16
Yes	7	33.3		0	0		3	12.5	
Type of practice									
Joint practice	15	71.4	0.90	1	100	0.56	17	70.8	0.99
Other practice	5	23.8		0	0		6	25	
Location of the practice									
Large city	3	14.3	0.19	0	0	0.54	6	25	0.70
Other locations	18	85.7		1	100		17	70.8	

**Fig. 1:**
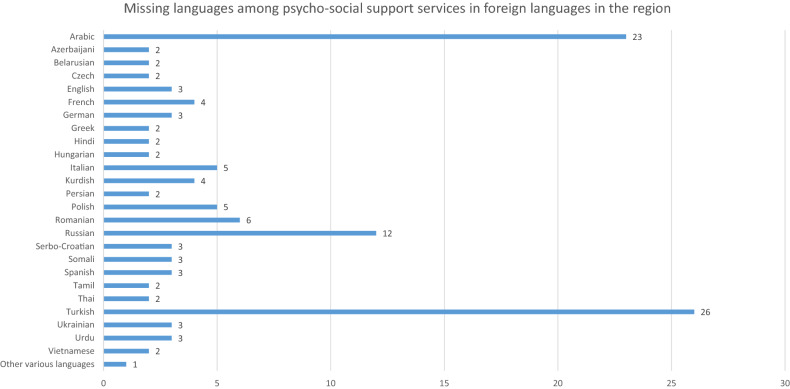
Number of haematologists and oncologist reporting the lack of psycho-social support services in each specific foreign language in their region (*N* = 55)

Nearly half of the doctors (26 [47%], and 23 [42%]) reported missing psycho-social services in Turkish and Arabic, respectively, in their region. Other commonly reported missing languages were: Russian (12 doctors), Romanian (6 doctors), Italian and Polish (5 doctors each) (Fig. 1).

### Group comparisons

Doctors with less than 20 years of work experience referred patients more often to psycho-social support services hosted in patients’ associations (75% vs 25%; *p* = 0.02), than doctors with more years of work experience.

Doctors working in practices in large cities referred patients less often to psycho-social support services in cancer counselling centres than doctors working in medium-to-smaller cities or villages (12% vs 88%; *p* = 0.01) (Table 4).

**Table 4:** Referral to the various psycho-social support services by socio-demographic characteristics of the doctors and characteristics of the practices they work in. Differences between groups tested with Pearson's chi-squared tests (*p*-values reported) (*N* = 55)

Covariates	Outpatient Psychotherapy			Psycho-social cancer counselling centre			Self-help group			Patients’ association				Outpatient clinics				Other services		
	Yes		*p*	Yes		*p*	Yes		*p*	Yes		*p*		Yes			*p*	Yes		*p*
	*N*	%		*N*	%		*N*	%		*N*	%			*N*		%		*N*	%	
Gender																				
Female	6	20.7	0.45	9	27.3	0.60	4	30.8	0.54	2	50	0.27		2		50	0.27	0	0	0.21
Male	21	72.4		22	66.7		8	61.5		2	50			2		50		4	100	
Years of age																				
< 60 years	19	65.5	0.55	22	66.7	0.36	7	53.9	0.50	4	100	0.10		4		100	0.10	3	75	0.57
≥ 60 years	10	34.5		11	33.3		6	46.2		0	0			0		0		1	25	
Years of work experience																				
< 20 years	6	20.7	0.39	9	27.3	0.70	4	30.8	0.61	3	75	0.02		2		50	0.02	0	0	0.22
≥ 20 years	23	79.3		24	72.7		9	69.2		1	25			2		50		4	100	
Country of birth																				
Germany	23	79.3	0.17	29	87.9	0.53	10	76.9	0.32	3	75	0.54		3		75	0.54	4	100	0.39
Other/Missing	6	20.7		4	12.1		3	23.1		1	25			1		25		0	0	
Psycho-oncological further education																				
No	21	72.4	0.97	23	69.7	0.05	7	53.9	0.19	4	100	0.27		4		100	0.27	3	75	0.88
Yes	6	20.7		10	30.3		4	30.8		0	0			0		0		1	25	
Type of practice																				
Joint practice	23	79.3	0.17	21	63.6	0.06	8	61.5	0.48	4	100	0.22		2		50	0.22	4	100	0.22
Other practice	5	17.2		11	33.3		4	30.8		0	0			2		50		0	0	
Location of the practice																				
Large city	9	31	0.09	4	12.1	0.01	2	15.4	0.55	2	50	0.18		1		25	0.92	1	25	0.92
Other locations	19	65.5		29	87.9		10	76.9		2	50			3		75		3	75	

Male doctors were more satisfied with the network with the providers of psycho-social support services than female doctors (mean score = 2.8 vs 2.2; *p* = 0.05) (Table 5).

**Table 5:** Satisfaction with network, organization, and cooperation with psycho-social support service providers by socio-demographic characteristics of the doctors and characteristics of the practices they work in. Differences between groups tested using Kruskal-Wallis (*p*-values reported) (*N* = 55)

Covariates	Satisfaction with network				Satisfaction with organization				Satisfaction with cooperation			
	*N*	Mean	Std	*p*	*N*	Mean	Std	*p*	*N*	Mean	Std	*p*
Gender												
Female	13	2.2	0.8	0.05	12	2.1	1.0	0.47	11	2.2	1.0	0.20
Male	34	2.8	1.1		35	2.4	1.1		35	2.7	1.1	
Years of age												
< 60 years	33	2.6	1.0	0.59	33	2.3	1.1	0.71	32	2.5	1.1	0.84
≥ 60 years	18	2.4	1.1		18	2.2	1.0		17	2.5	1.1	
Years of work experience												
< 20 years	13	2.5	0.9	0.70	13	2.2	1.1	0.68	13	2.3	1.0	0.42
≥ 20 years	38	2.6	1.1		38	2.3	1.1		36	2.6	1.1	
Country of birth												
Germany	45	2.6	1.0	0.34	45	2.3	1.1	0.12	45	2.5	1.1	0.65
Other/Missing	6	2.2	1.2		6	1.7	1.2		4	2.8	1.0	
Psycho-oncological further education												
No	38	2.5	1.0	0.25	38	2.2	1.1	0.55	38	2.5	1.0	0.36
Yes	11	2.9	1.0		11	2.5	1.1		10	2.8	1.1	
Type of practice												
Joint practice	36	2.6	1.2	0.51	37	2.3	1.2	0.47	36	2.6	1.2	0.38
Other practice	13	2.4	0.8		12	2.0	0.9		12	2.3	0.8	
Location of the practice												
Large city	12	2.5	1.0	0.83	11	2.0	1.0	0.40	11	2.3	1.0	0.42
Other locations	38	2.6	1.1		39	2.3	1.1		38	2.6	1.1	

## Discussion

We aimed at investigating the experience of office-based haematologists and oncologists with outpatient psycho-social support services in Germany.

Doctors working in larger cities reported referring their patients less often to psycho-social services hosted in cancer counselling centres, than doctors working in small and middle-large cities. This result might be attributed to the lower presence of these services in larger cities. Singer et al. (2012) reported that half of the counselling centres in the German federal state Sachsen were in middle-large cities, while only around one-third and one-sixth of these centres were in smaller or larger cities, respectively. Furthermore, these services often have barriers in accessibility (Giesler et al. 2015; Singer et al. 2012). Hence, oncologists in larger cities might be reticent in referring their patients to these services. This was also observed by Hempler et al. (2021b): doctors reported that they refer their patients more easily to centres providing psycho-social support services, which were in close proximity to the practice.

Doctors with less than 20 years of work experience referred more often to psycho-social support services hosted by patients’ associations than doctors with more than 20 years of work experience. Speculation might be drawn on whether younger doctors have more information on alternative providers of psycho-social support services, compared to older doctors. Another potential explanation might be that because of their fewer years of work experience, younger doctors did not yet build a network of personal contacts with psychologists or social workers providing psycho-social support services. According to Hempler et al. (2021b), doctors considered the referral to psycho-social support services to be a complicated process and, therefore, they heavily rely on personal connections with the providers of the services. The location might also play a role: younger doctors might be more present in larger cities, and, therefore, be confronted with the aforementioned lacks of services.

Female doctors were less satisfied with their network with providers of psycho-social support services than male doctors. To the knowledge of the authors, no previous study reports similar results. However, it has been documented that women—in this case patients—have a more positive attitude towards psycho-social support services, due to a lower perception of stigmatization around psycho-social help seeking (Eichler et al. 2019; Faller et al. 2017; Steginga et al. 2008). Speculations can be drawn that female doctors might perceive lower stigmatization in recommending these services. In this scenario, female oncologists might consider the services more valuable and, thus, be more prone to offer it to patients in need. These larger volume of requests, could in turn lead to higher exposure to structural issues, e.g. logistic and organizational difficulties.

More than one doctor in three (38%) reported that psycho-social services are not sufficient in their region. Regarding psycho-social support services in foreign languages, the large majority (80%) of the doctors reported that these services are unavailable. This lack in psycho-social support services in foreign languages was previously reported by Schultz et al. (2018).

### Implications for future research

Further research could aim to look at the experiences with outpatient psycho-social support services among oncologists in Germany on a regional level. Moreover, the specific local concentration of patients with cancer and different migration backgrounds could be also considered. In addition, interventions leading to better network between oncologists and psycho-social support services’ providers could be investigated. Finally, further research could look at overcoming the limitations of this study. More in detail, beside the small sample size, we included only office-based haematologists and oncologists. As psycho-social needs of cancer patients are known to vary based on the type of cancer (Krebber et al. 2014; Singer et al. 2009), a wider research including different oncological specializations should be conducted.

### Implications for policy makers and service providers

Oncologists (and their patients) should be provided with available and easy-to-reach psycho-social support services in their region. Special efforts should be made to provide services in foreign languages or—at least—access to linguistically and culturally competent translators for psycho-social support services.

### Limitations

This study was considered of interest as doctors’ recommendation is among the most important predictors of participation in psycho-social support services (Eakin and Strycker 2001; Frey Nascimento et al. 2019). Hence, a positive experience for the oncologists in terms of availability of the services and cooperation with the providers could enhance the recommendations and the participation of the patients.

The generalization of the findings of this study is limited due to the small study sample, which does not only limit the results per se but, by forcing to build aggregated groups, also disallowed for multivariate comparisons. Moreover, the definition of psycho-social support services for patients with migration background was based on the language in which the service could be provided. Therefore, no evaluation is present on whether these services could still present access barriers, e.g. lack of cultural competency. Furthermore, the participating haematologists and oncologists shared the same specialization in outpatient care and the same network. Hence, the results should be only referred to office-based haematologists and oncologists.

### Conclusions

Office-based haematologists and oncologists in Germany reported a non-sufficient availability of psycho-social support services. Hence, a higher regional availability of psycho-social support services should be considered, both in terms of overall services and especially of services for non-German speaking patients. Moreover, a greater focus should be placed on services in Turkish and Arabic, followed by Russian, Romanian, Italian, and Polish. The doctors’ work experience and gender, as well as the city they work in were associated with different experiences with psycho-social support services. This aspect should be considered when developing strategies in support of referral to psycho-social support services. These results should be evaluated in the explorative nature of this study and—thus—when generalizing them, awareness must be present regarding the limitations in terms of study design and study population.

## Data Availability

The data that support the findings of this study are available on request from the corresponding author. The data are not publicly available due to privacy or ethical restrictions.

## References

[CR1] Buffart LM et al (2020) Effects and moderators of coping skills training on symptoms of depression and anxiety in patients with cancer: aggregate data and individual patient data meta-analyses. Clin Psychol Rev 80:101882. 10.1016/j.cpr.2020.101882 32640368 10.1016/j.cpr.2020.101882

[CR2] Eakin EG, Strycker LA (2001) Awareness and barriers to use of cancer support and information resources by HMO patients with breast, prostate, or colon cancer: patient and provider perspectives Psycho-Oncol 10:103–113. 10.1002/pon.500 10.1002/pon.500 11268137

[CR3] Eichler M et al. (2019) Use of psychosocial services by lung cancer survivors in Germany: Results of a German multicenter study (LARIS) Strahlentherapie und Onkologie: Organ der Deutschen Rontgengesellschaft [et al] 195:1018–1027. 10.1007/s00066-019-01490-1 10.1007/s00066-019-01490-1 31292665

[CR4] Faller H et al (2017) Utilization of professional psychological care in a large German sample of cancer patients. Psychooncology 26:537–543. 10.1002/pon.4197 27327213 10.1002/pon.4197

[CR5] Frey Nascimento A et al (2019) Oncologist recommendation matters!—predictors of psycho-oncological service uptake in oncology outpatients. Psychooncology 28:351–357. 10.1002/pon.4948 30466146 10.1002/pon.4948

[CR6] Giesler J et al (2015) Ambulante psychoonkologische Versorgung durch Krebsberatungsstellen—Leistungsspektrum und Inanspruchnahme durch Patienten und Angehörige Psychother Psychosom. Med Psychol 65:450–458 10.1055/s-0035-1554718 26200246

[CR7] Goodwin PJ et al (2001) The effect of group psychosocial support on survival in metastatic breast cancer. N Engl J Med 345:1719–1726. 10.1056/NEJMoa011871 11742045 10.1056/NEJMoa011871

[CR8] Hempler I, Heidt V, Riccetti N, Singer S, Hermes-Moll K (2021a) Wir wussten, dass es schwierig wird, doch damit haben wir nicht gerechnet! Erfahrungen im Studieneinschluss von Krebspatient*innen mit Migrationshintergrund und Angehörigen im Bereich der Psychoonkologie Zeitschrift für Evidenz. Fortbildung Und Qualität Im Gesundheitswesen. 10.1016/j.zefq.2021.08.002 10.1016/j.zefq.2021.08.002 PMC8405365 34474995

[CR9] Hempler I, Riccetti N, Hermes-Moll K, Heidt V, Singer S (2021b) Psychoonkologische Versorgung von Menschen mit Migrationshintergrund und ihren Angehörigen—Ergebnisse aus leitfadengestützten Einzelinterviews mit Ärztinnen und Ärzten Psychother Psychosom. Med Psychol 71:335–342 10.1055/a-1390-4061 33773520

[CR10] Hempler I, Riccetti N, Kalie L, Heidt V, Singer S, Hermes-Moll K (2021c) Es war zu viel, zu früh, zu fremd. Onkologe 27:1233–1240. 10.1007/s00761-021-01043-1

[CR11] Hermes-Moll K et al (2022a) Ansprache sensibler Themen im ärztlichen Gespräch mit übersetzenden Angehörigen bei der Identifikation psychoonkologischer Versorgungsbedarfe von Patient*innen mit Migrationshintergrund [Addressing sensitive topics in medical conversations that require translation by relatives when identifying the psycho-oncological care needs of patients with a migrant background]. Forum 30:1–5. 10.1007/s12312-022-01100-9

[CR12] Hermes-Moll K et al (2022b) Empfehlungen zur psychosozialen und psychoonkologischen Versorgung. InFo Hämatologie + Onkologie 25(5):46–55. 10.1007/s15004-022-9031-5

[CR13] Herschbach P, Mandel T (2011) Psychoonkologische Versorgung Im Nationalen Krebsplan. Onkologe 17:1107–1114. 10.1007/s00761-011-2149-y

[CR14] Kalter J et al (2018) Effects and moderators of psychosocial interventions on quality of life, and emotional and social function in patients with cancer: an individual patient data meta-analysis of 22 RCTs. Psychooncology 27:1150–1161. 10.1002/pon.4648 29361206 10.1002/pon.4648 PMC5947559

[CR15] Kowalski C, Ferencz J, Singer S, Weis I, Wesselmann S (2016) Frequency of psycho-oncologic and social service counseling in cancer centers relative to center site and hospital characteristics: Findings from 879 center sites in Germany, Austria, Switzerland, and Italy. Cancer 122:3538–3545. 10.1002/cncr.30202 27481151 10.1002/cncr.30202

[CR16] Krebber AM et al (2014) Prevalence of depression in cancer patients: a meta-analysis of diagnostic interviews and self-report instruments. Psychooncology 23:121–130. 10.1002/pon.3409 24105788 10.1002/pon.3409 PMC4282549

[CR18] Marchioro G et al (1996) The impact of a psychological intervention on quality of life in non-metastatic breast cancer. Eur J Cancer 32:1612–1615. 10.1016/0959-8049(96)00134-7 10.1016/0959-8049(96)00134-7 8911127

[CR19] Riccetti N, Werner AM, Ernst M, Hempler I, Singer S (2020) Informations- und Unterstützungsbedürfnisse krebskranker Migrant*innen und ethnischer Minderheiten – ein Umbrella-Review. Onkologe 26:957–965. 10.1007/s00761-020-00827-1

[CR20] Riccetti N et al (2022a) Financial difficulties in breast cancer survivors with and without migration background in Germany – results from the prospective multicentre cohort study BRENDA II. Support Care Cancer 30:6677–6688. 10.1007/s00520-022-07074-7 35507113 10.1007/s00520-022-07074-7 PMC9213307

[CR21] Riccetti N et al (2022b) Linguistic barriers with migrant cancer patients and their relatives in Germany: an explorative article on the perspective of the oncologists from the mixed-methods study POM. Res Health Serv Reg 1:3. 10.1007/s43999-022-00001-7 39177829 10.1007/s43999-022-00001-7 PMC11264866

[CR22] Schulz H et al. (2018) Psychoonkologische Versorgung in Deutschland: Bundesweite Bestandsaufnahme und Analyse—Wissenschaftliches Gutachten im Auftrag des Bundesministeriums für Gesundheit. Universitätsklinikum Hamburg-Eppendorf Hamburg

[CR23] Singer S et al (2009) Hospital anxiety and depression scale cutoff scores for cancer patients in acute care. Br J Cancer 100:908–912. 10.1038/sj.bjc.6604952 19240713 10.1038/sj.bjc.6604952 PMC2661775

[CR24] Singer S, Bretschneider N, Lehmann-Laue A, Schröter K, Porzig R, Frenschkowski S, Riedel SJDG (2012) Psychosoziale Krebsberatungsstellen–eine Analyse der Versorgungsrealität in Sachsen. Das Gesundheitswesen 74:736–741 22012562 10.1055/s-0031-1285899

[CR25] Singer S et al (2013a) Co-morbid mental health conditions in cancer patients at working age—prevalence, risk profiles, and care uptake. Psychooncology 22:2291–2297. 10.1002/pon.3282 23494948 10.1002/pon.3282

[CR26] Singer S, Dieng S, Wesselmann S (2013b) Psycho-oncological care in certified cancer centres—a nationwide analysis in Germany. Psychooncology 22:1435–1437. 10.1002/pon.3145 22855347 10.1002/pon.3145

[CR27] Singer S et al (2022) Awareness and use of psychosocial care among cancer patients and their relatives—a comparison of people with and without a migration background in Germany. J Cancer Res Clin Oncol. 10.1007/s00432-022-04091-1 35689688 10.1007/s00432-022-04091-1 PMC9188276

[CR28] Steginga SK, Campbell A, Ferguson M, Beeden A, Walls M, Cairns W, Dunn J (2008) Socio-demographic, psychosocial and attitudinal predictors of help seeking after cancer diagnosis. Psychooncology 17:997–1005. 10.1002/pon.1317 18203243 10.1002/pon.1317

[CR29] Sze M et al (2015) Migrant health in cancer: outcome disparities and the determinant role of migrant-specific variables. Oncologist 20:523–531. 10.1634/theoncologist.2014-0274 25802406 10.1634/theoncologist.2014-0274 PMC4425378

[CR30] Tibubos AN et al (2018) Is assessment of depression equivalent for migrants of different cultural backgrounds? Results from the German Population-Based Gutenberg Health Study (GHS). Depress Anxiety 35:1178–1189. 10.1002/da.22831 30156742 10.1002/da.22831

[CR31] Warth M, Zöller J, Köhler F, Aguilar-Raab C, Kessler J, Ditzen B (2020) Psychosocial interventions for pain management in advanced cancer patients: a systematic review and meta-analysis. Curr Oncol Rep 22:3. 10.1007/s11912-020-0870-7 31965361 10.1007/s11912-020-0870-7 PMC8035102

[CR32] Zeissig SR, Singer S, Koch L, Blettner M, Arndt VJP-PPMP (2015) Inanspruchnahme psychoonkologischer Versorgung im Krankenhaus und in Krebsberatungsstellen durch Brust- Darm-Und Prostatakrebsüberlebende. PPmP-Psychotherapie· Psychosomatik·medizinische Psychologie 65:177–182 10.1055/s-0034-1395627 25485601

